# Prognostic utility of biopsy-based *PTEN* and *ERG* status on biochemical progression and overall survival after SBRT for localized prostate cancer

**DOI:** 10.3389/fonc.2024.1381134

**Published:** 2024-03-22

**Authors:** Michael C. Repka, Tamir Sholklapper, Alan L. Zwart, Malika Danner, Marilyn Ayoob, Thomas Yung, Siyuan Lei, Brian T. Collins, Deepak Kumar, Simeng Suy, Ryan A. Hankins, Amar U. Kishan, Sean P. Collins

**Affiliations:** ^1^ Department of Radiation Oncology, University of North Carolina (UNC) School of Medicine, Chapel Hill, NC, United States; ^2^ Department of Radiation Medicine, Georgetown University Hospital, Washington, DC, United States; ^3^ Department of Radiation Oncology, Tampa General Hospital, Tampa, FL, United States; ^4^ Julius L Chambers Research Institute, North Carolina Central University, Durham, NC, United States; ^5^ Department of Urology, Georgetown University Hospital, Washington, DC, United States; ^6^ Department of Radiation Oncology, University of California, Los Angeles (UCLA) Health, Los Angeles, CA, United States

**Keywords:** prostate cancer, PTEN, ERG, SBRT, radiotherapy

## Abstract

**Introduction/background:**

Phosphatase and tensin homolog (PTEN) genomic deletions and transmembrane protease, serine 2/v-ets avian erthyroblastosis virus E26 oncogene homolog (ERG) rearrangements are two of the most common genetic abnormalities associated with prostate cancer. Prior studies have demonstrated these alterations portend worse clinical outcomes. Our objective is to evaluate the impact of biopsy-determined PTEN losses and TMPRSS2-ERG fusion on biochemical progression-free survival (bPFS) and overall survival (OS) in patients who receive SBRT for localized prostate cancer.

**Methods/materials:**

Patients received SBRT for localized prostate cancer on a prospective quality-of-life (QoL) and cancer outcomes study. For each patient, the single biopsy core with the highest grade/volume of cancer was evaluated for PTEN and ERG abnormalities. Differences in baseline patient and disease characteristics between groups were analyzed using ANOVA for age and χ^2^ for categorical groupings. bPFS and OS were calculated using the Kaplan Meier (KM) method with Log-Rank test comparison between groups. Predictors of bPFS and OS were identified using the Cox proportional hazards method. For all analyses, *p <*0.05 was considered statistically significant.

**Results:**

Ninety-nine consecutive patients were included in the analysis with a median follow-up of 72 months. A statistically significant improvement in bPFS (*p =* 0.018) was observed for wild type ERG patients with an estimated 5-year bPFS of 94.1% vs. 72.4%. Regarding PTEN mutational status, significant improvements in were observed in both bPFS (p = 0.006) and OS (p < 0.001), with estimated 5-year bPFS rates of 91.0% vs. 67.9% and 5-year OS rates of 96.4% vs. 79.4%. When including both ERG and PTEN mutational status in the analysis, there were statistically significant differences in both bPFS (p = 0.011) and OS (p < 0.001). The estimated 5-year bPFS rates were 100%, 76.6%, 72.9%, and 63.8% for patients with ERG+/PTEN+, ERG-/PTEN+, ERG+/PTEN-, and ERG-/PTEN- phenotypes respectively. The estimated 5-year OS rates were 93.9%, 100%, 80.0%, and 78.7% for patients with ERG+/PTEN+, ERG-/PTEN+, ERG+/PTEN-, and ERG-/PTEN- phenotypes respectively.

**Conclusion:**

ERG rearrangements and PTEN deletions detected on biopsy samples are associated with poorer oncologic outcomes in prostate cancer patients treated with SBRT and merit further study in a dedicated prospective trial.

## Introduction

Prostate cancer is the second most frequently diagnosed cancer in the United States, and the most common malignant disease diagnosed in males (1). In patients with localized prostate cancer, choice of treatment can be difficulty given the multiple options available, including radical prostatectomy, radiation therapy, active surveillance, in addition to newer, experimental focal therapies (2). Further complicating the issue for patients who elect to pursue radiotherapy is the question of whether and for how long to incorporate concomitant androgen deprivation therapy (ADT).

Risk stratification for patients with prostate cancer has traditionally used a set of clinico-pathologic features, such as Gleason grade group, serum PSA level, and tumor staging as determined by digital rectal examination (DRE). These features have been incorporated into consensus guidelines in order to stratify patients and help guide treatment decisions (3). However, this system remains imprecise and may be limited by multiple factors including variable inter-rater reliability, comorbidities such as benign prostatic hyperplasia (BPH), and poor sensitivity. In other disease sites, tumor genetics analyses are frequently employed to overcome the limitations of standard staging algorithms (4). In patients with breast cancer for example, analysis of tumor genetics is endorsed by national consensus guidelines, widespread in clinical practice, and frequently impacts choice of treatment. A growing body of evidence suggests that genetic analyses in patients with prostate cancer may improve prognostication, but overall their role remains uncertain (5).

Phosphatase and tensin homolog (*PTEN*) genomic deletions and transmembrane protease, serine 2/v-ets avian erthyroblastosis virus E26 oncogene homolog (*ERG*) rearrangements are two of the most common genetic abnormalities associated with prostate cancer. Prior studies have revealed that changes in wild-type PTEN and ERG portend worse clinical outcomes (6, 7). While many early studies were performed on whole-gland samples after radical prostatectomy, subsequent biopsy-based analyses have led to their inclusion in several genomic assays and prostate cancer outcome scores. However, evaluations of therapeutic response to radiotherapy are quite heterogenous. In the present analysis our objective is to evaluate the impact of biopsy-determined *PTEN* losses and *TMPRSS2-ERG* fusion on biochemical progression-free survival (bPFS) and overall survival (OS) in patients who receive SBRT for localized prostate cancer.

## Methods & materials

### Patients & treatments

Patients included in this single-institution analysis received SBRT for localized prostate cancer and are followed on a prospective quality-of-life (QoL) and cancer outcomes study. Patients included in the analysis had clinically localized prostate cancer and were treated with SBRT, either as monotherapy or in conjunction with more comprehensive, conventionally fractionated intensity-modulated radiotherapy (IMRT). Patients received ADT on an individual basis following shared decision making (SDM) with their radiation oncologist and urologist. Patients were excluded if they received surgery, brachytherapy, or other form of local therapy as part of their cancer care. Patients with clinical N1 disease (AJCC 8^th^ Ed. Stage IVA) were excluded. Prior transurethral resection of the prostate (TURP), hip replacement, or contraindications to MRI were not considered exclusion criteria.

SBRT was performed as has been previously described in multiple prior reports (8, 9). Briefly, patients treated with SBRT one received a dose of 3500 cGy – 3625 cGy in five fractions delivered over 5 – 11 days. In patients treated with combined IMRT and SBRT (10), three fractions of 650 cGy each (1950 cGy total) were delivered prior to IMRT (4500 cGy in 25 fractions of 180 cGy each). All patients had permanent fiducial markers placed within the prostate prior to any radiotherapy treatment. Patients were followed at 1 month, 3 months, 6 months, 9 months, 12 months in the first year following treatment, then every 6 months until 5 years, then annually thereafter. Clinical evaluation including patient-reported quality-of-life, physician-graded toxicity, digital rectal examination, serum PSA, and serum testosterone was performed at each visit. Biochemical progression-free survival (bPFS) was determined using the Phoenix criteria (PSA nadir + 2 ng/mL).

### Pathology & genetic analyses

For each patient, the single biopsy core with the highest grade/volume of cancer was evaluated for PTEN and ERG abnormalities. Testing was performed using the commercially available ProstaVysion test (Bostwick Laboratories, Glen Allen, VA). Patients were considered to have PTEN abnormality with either heterozygous or homozygous gene deletions.

### Statistical analysis

Differences in baseline patient and disease characteristics between groups were analyzed using ANOVA for age and χ^2^ for categorical groupings. bPFS and OS were calculated using the Kaplan Meier (KM) method with Log-Rank test comparison between groups. Predictors of bPFS and OS were identified using the Cox proportional hazards method. Significant predictors of OS on univariable analysis were incorporated into a multivariable Cox proportional hazards model. Due to the large number of statistically significant predictors of bPFS in relation to the number of events, multivariable analysis was not performed (11).For all analyses, *p <*0.05 was considered statistically significant. Statistical analyses were performed using SPSS version 28.0.0.0 (IBM Corporation, Armonk, NY).

## Results

Ninety-seven consecutive patients were included in the analysis with a median follow-up of 72 months. The median patient age was 69.6 years (range 53 – 85 years). Forty-three patients (44.3%) were Black, forty-six (47.4%) were white, with a variety of other ethnicities comprising the remaining 8.2% of patients. In general, the cohort was relatively healthy, with nearly 90% of patients having a Charlson Comorbidity Index of 0-1. In regards to oncologic characteristics, a majority of patients (60.8%) presented with a PSA less than 10 ng/mL at baseline, and a minority of patients were found to have evidence of extra-prostatic disease with 4 patients (4.0%) diagnosed with clinical T3 disease. Furthermore, there was relatively even distribution of Gleason grade groups observed in this study; the most frequently observed was grade 2, comprising only 33.0% of the cohort. Patients were similarly well distributed by NCCN Risk Groups, with 35.4% of patients in the low or favorable intermediate risk groups, 32.3% of patients in the unfavorable intermediate risk group, 20.2% of patients in the high risk group, and 12.1% of patients in the very high risk group. The majority of patients (79.4%) were treated with SBRT to the prostate and proximal seminal vesicles alone, while the remainder received conventionally fractionated pelvic radiotherapy in conjunction with a three-fraction SBRT boost. Slightly more than half of patients (57.7%) received ADT in conjunction with radiotherapy.

Clinical and treatment characteristics were reasonably well balanced when stratified by ERG and PTEN mutational status. Patients with ERG mutated phenotypes were more statistically more likely to present with higher T-stage disease, and there were trends toward higher Gleason grade, higher NCCN risk grouping, and increased ADT usage in the patients with an ERG mutation. Similarly, while significant differences between the groups were identified when stratified by PTEN mutational status, similar trends were noted with regard to Gleason grade, T-stage, NCCN risk grouping, and ADT usage. Full baseline characteristics are available in **Table 1**.

**Table 1:** Baseline Characteristics for all patients, as well as stratified by ERG and PTEN mutational status.

		All		ERG					PTEN				
				wild type		mutant		p	wild type		mutant		p
		Median	Range	Median	Range	Median	Range		Median	Range	Median	Range	
**Age (years)**		69.6	53 - 85	69.8	55 - 82	69.2	53 - 85	0.657	68.55	53 - 85	70.1	62 - 80	0.117
		**n**	**%**	**n**	**%**	**n**	**%**		**n**	**%**	**n**	**%**	
**Ethnicity**	**Black**	43	44.3%	28	49.1%	15	39.5%	0.333	31	48.4%	11	40.7%	0.412
	**Other**	8	8.2%	6	10.5%	2	5.3%		6	9.4%	1	3.7%	
	**White**	46	47.4%	23	40.4%	21	55.3%		27	42.2%	15	55.6%	
**CCI**	**0**	62	67.0%	38	70.4%	22	61.1%	0.726	41	68.3%	15	57.7%	0.615
	**1**	21	22.3%	11	20.4%	10	27.8%		13	21.7%	8	30.8%	
	**2**	9	10.6%	5	9.3%	4	11.1%		6	10.0%	3	11.5%	
	**Unknown**	5											
**T Stage**	**T1c**	58	59.8%	39	68.4%	18	47.4%	**0.012**	42	65.6%	12	44.4%	0.304
	**T2a**	20	20.6%	11	19.3%	9	23.7%		11	17.2%	8	29.6%	
	**T2b/c**	15	15.5%	6	10.5%	9	23.7%		9	14.1%	6	22.2%	
	**T3**	4	4.1%	1	1.8%	2	5.3%		2	3.1%	1	3.7%	
**PSA (ng/mL)**	**<10**	59	60.8%	33	57.9%	25	65.8%	0.808	37	57.8%	19	70.4%	0.449
	**10-20**	26	26.8%	17	29.8%	8	21.1%		17	26.6%	6	22.2%	
	**20+**	12	12.4%	7	12.3%	5	13.2%		10	15.6%	2	7.4%	
**PSA Density (ng/mL/mL)**	**<0.15**	24	24.7%	13	22.8%	10	26.3%	0.654	15	23.4%	7	25.9%	0.8
	**0.15+**	73	75.3%	44	77.2%	28	73.7%		49	76.6%	20	74.1%	
**Grade**	**1**	24	24.7%	15	26.3%	8	21.1%	0.844	18	28.1%	3	11.1%	0.107
	**2**	32	33.0%	19	33.3%	13	34.2%		20	31.3%	12	44.4%	
	**3**	18	18.6%	11	19.3%	7	18.4%		10	15.6%	8	29.6%	
	**4/5**	23	23.7%	12	21.1%	10	26.3%		16	25.0%	4	14.8%	
**NCCN RG**	**LIR/FIR**	35	35.4%	22	38.6%	12	31.6%	0.229	26	40.6%	6	22.2%	0.289
	**UIR**	32	32.3%	18	31.6%	14	36.8%		19	29.7%	13	48.1%	
	**HR**	20	20.2%	14	24.6%	6	15.8%		13	20.3%	5	18.5%	
	**VHR**	12	12.1%	3	5.3%	6	15.8%		6	9.4%	3	11.1%	
**Dose (cGy)**	**3500**	40	41.2%	25	43.9%	15	39.5%	0.744	25	39.1%	13	48.1%	0.716
	**3625**	37	38.1%	21	36.8%	15	39.5%		26	40.6%	9	33.3%	
	**1950**	20	20.6%	11	19.3%	8	21.1%		13	20.3%	5	18.5%	
**ADT**	**Yes**	56	57.7%	36	63.2%	20	52.6%	0.148	40	62.5%	14	51.9%	0.345
	**No**	41	42.3%	21	36.8%	18	47.4%		24	37.5%	13	48.1%	

Range represents the full range of data represented in the study rather than the interquartile range (IQR). CCI (Charlson Comorbidity Index), NCCN RG (National Comprehensive Cancer Network Risk Group), ADT (Androgen Deprivation Therapy), LR (Low Risk), FIR (Favorable Intermediate Risk), UIR (Unfavorable Intermediate Risk), HR (High Risk).

**Figure 1:**
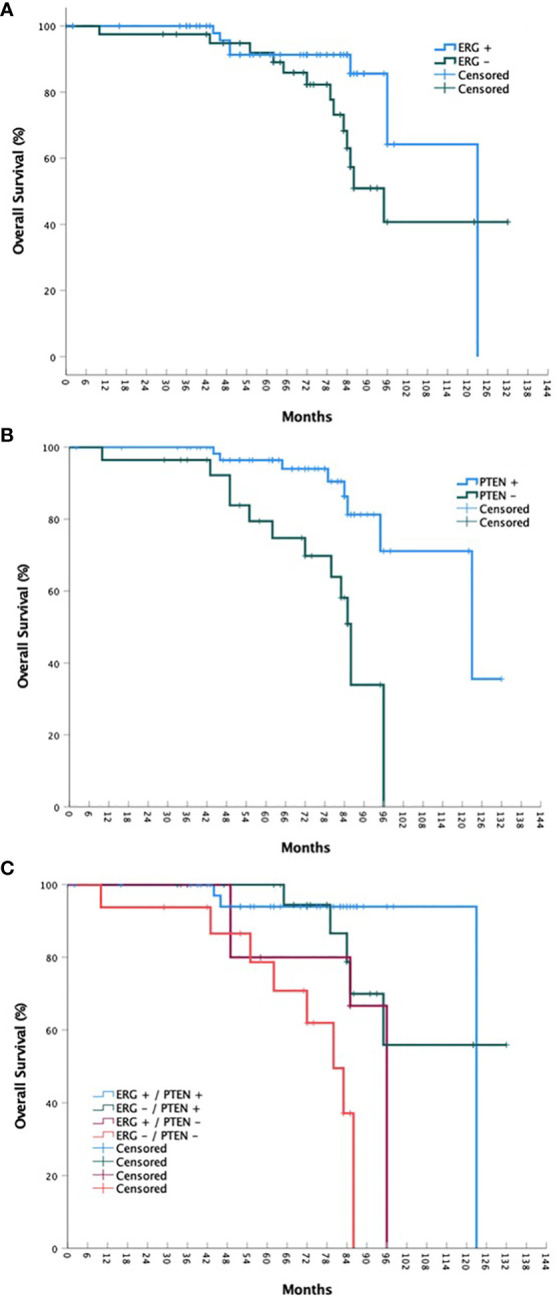
bPFS stratified by ERG mutational status **(A)**, PTEN mutational status **(B)**, and ERG/PTEN mutational status **(C)**.

**Figure 2:**
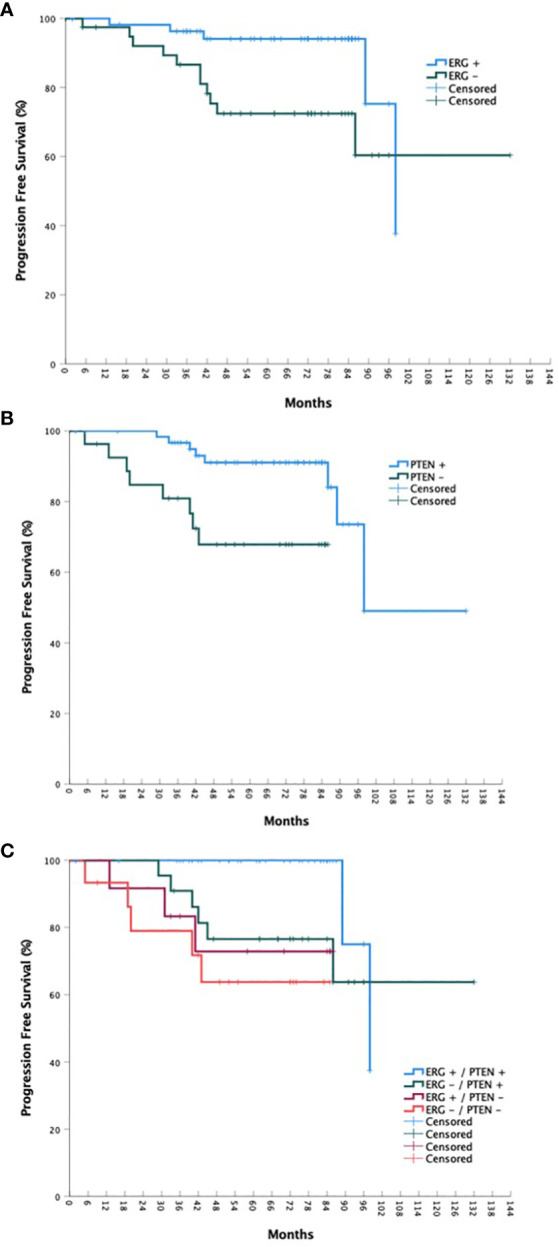
OS stratified by ERG mutational status **(A)**, PTEN mutational status **(B)**, and ERG/PTEN mutational status **(C)**.

For the overall cohort, the estimated 5-year bPFS was 85.2% with a median bPFS of 98 months, while the estimated 5-year OS was 91.8% with a median OS of 123 months (**Figures 1** and **2**). When stratified by ERG mutational status, a statistically significant improvement in bPFS (*p =* 0.018) was observed for wild type patients with an estimated 5-year bPFS of 94.1% vs. 72.4%. There was a strong trend towards improvement in overall survival as well, though this was not statistically significant. When stratified by PTEN mutational status, significant improvements were observed in both bPFS (p = 0.006) and OS (p < 0.001), with estimated 5-year bPFS rates of 91.0% vs. 67.9% and 5-year OS rates of 96.4% vs. 79.4%. When including both ERG and PTEN mutational status in the analysis, there were statistically significant differences in both bPFS (p = 0.011) and OS (p < 0.001). The estimated 5-year bPFS rates were 100%, 76.6%, 72.9%, and 63.8% for patients with ERG+/PTEN+, ERG-/PTEN+, ERG+/PTEN-, and ERG-/PTEN- phenotypes respectively. The estimated 5-year OS rates were 93.9%, 100%, 80.0%, and 78.7% for patients with ERG+/PTEN+, ERG-/PTEN+, ERG+/PTEN-, and ERG-/PTEN- phenotypes respectively.

On univariable analysis, factors associated with worse biochemical PFS were a CCI of 1 (vs. 0; HR 7.022, 95% CI 2.108 – 23.392, p = 0.002, clinical T2b/c disease (vs. T1c, HR 5.011, 95% CI 1.332 – 18.851, p = 0.017), high risk status (vs. low/favorable intermediate risk, HR 8.919, 95% CI 1.674 – 47.527, p = 0.02), very high risk status (HR 6.448, 95% CI 1.011 – 41.112, p = 0.049), grade group 3 disease (vs. grade group 1/2, HR 4.353, 95% CI 1.157 – 16.374, p = 0.030), grade group 4/5 disease (HR 4.235, 95% CI 1.182 – 15.174, p = 0.027), ERG+/PTEN- status (vs. ERG+/PTEN+, HR 8.272, 95% CI 1.303 – 52.516, p = 0.025), and ERG-/PTEN- status (HR 13.66, 95% CI 2.444 – 76.364, p = 0.003). In regards to overall survival, factors associated with poorer outcomes included a CCI of 1 (HR 3.764, 95% CI 1.201 – 11.800, p = 0.023) or 2 (HR 14.095, 95% CI 3.719 – 53.421, p < 0.001), high risk status (HR 5.679, 95% CI 1.408 – 22.907, p = 0.015), and ERG-/PTEN- status (HR 9.684, 95% CI 2.385 – 39.323, p = 0.001). Full results are available in **Table 2**. On multivariable analysis, only CCI and ERG/PTEN status were associated with overall survival outcomes (**Table 3**).

**Table 2:** Univariable Cox Proportional Hazards Analysis for Progression Free Survival (PFS) and Overall Survival (OS).

	PFS			OS		
Variable	Hazard Ratio	95% CI	p	Hazard Ratio	95% CI	p
Ethnicity						
Non-White	(Ref)			(Ref)		
White	1.8	0.604 - 5.367	0.292	2.242	0.856 - 5.873	0.1
CCI						
0	(Ref)			(Ref)		
1	7.022	2.108 - 23.392	0.002	3.764	1.201 - 11.800	0.023
2	4.522	0.491 - 41.671	0.183	14.095	3.719 - 53.421	<0.001
T Stage						
T1c	(Ref)			(Ref)		
T2a	2.802	0.699 - 11.227	0.146	0.503	0.110 - 2.303	0.376
T2ab	5.011	1.332 - 18.851	0.017	1.231	0.385 - 3.938	0.726
T3+	3.1	0.345 - 27.880	0.313	2.301	0.497 - 10.660	0.287
PSA (ng/mL)						
<10	(Ref)			(Ref)		
10 - 20	1.061	0.319 - 3.530	0.923	1.243	0.416 - 3.718	0.697
20+	1.193	0.251 - 5.678	0.824	1.834	0.561 - 6.000	0.316
Grade						
1/2	(Ref)			(Ref)		
3	4.353	1.157 - 16.374	0.030	1.037	0.315 - 3.419	0.952
4/5	4.235	1.182 - 15.174	0.027	1.343	0.416 - 4.338	0.622
NCCN RG						
LR/FIR	(Ref)			(Ref)		
UIR	2.508	0.455 - 13.823	0.291	2.576	0.666 - 9.969	0.171
HR	8.919	1.674 - 47.527	0.01	5.679	1.408 - 22.907	0.015
VHR	6.448	1.011 - 41.112	0.049	2.137	0.356 - 12.831	0.406
ADT						
No	(Ref)			(Ref)		
Yes	2.773	0.928 - 8.288	0.068	2.363	0.912 - 6.125	0.077
ERG/PTEN						
+/+	(Ref)			(Ref)		
-/+	4.089	0.787 - 21.248	0.094	1.811	0.430 - 7.636	0.419
+/-	8.272	1.303 - 52.516	0.025	3.611	0.784 - 16.628	0.099
-/-	13.66	2.444 - 76.364	0.003	9.684	2.385 - 39.323	0.001

CCI (Charlson Comorbidity Index), NCCN RG (National Comprehensive Cancer Network Risk Group), ADT (Androgen Deprivation Therapy), LR (Low Risk), FIR (Favorable Intermediate Risk), UIR (Unfavorable Intermediate Risk), HR (High Risk), 95% CI (95% Confidence Interval).

**Table 3:** Multivariable Cox Proportional Hazards Analysis for Overall Survival (OS).

	OS		
Variable	Hazard Ratio	95% CI	p
CCI			
0	(Ref)		
1	2.202	0.563 - 8.604	0.56
2	12.098	2.301 - 63.616	0.003
NCCN RG			
LR/FIR	(Ref)		
UIR	0.756	0.147 - 3.889	0.756
HR	3.26	0.681 - 15.602	0.139
VHR	0.429	0.037 - 4.936	0.497
ERG/PTEN			
+/+	(Ref)		
-/+	0.999	0.191 - 5.212	0.999
+/-	6.58	1.154 - 37.512	0.034
-/-	15.522	2.414 - 99.791	0.004

CCI (Charlson Comorbidity Index), NCCN RG (National Comprehensive Cancer Network Risk Group), ADT (Androgen Deprivation Therapy), LR (Low Risk), FIR (Favorable Intermediate Risk), UIR (Unfavorable Intermediate Risk), HR (High Risk), 95% CI (95% Confidence Interval).

## Discussion

Since the 1998 publication of D’Amico et al’s landmark retrospective study (12), patients with localized prostate cancer have typically been sorted into low-, intermediate-, and high-risk cohorts. Although these risk groupings were not initially designed for the purpose of treatment selection, this clinical-pathologic stratification system has become the primary mode by which treatments are offered, particularly with regards to radiotherapy and incorporation of either elective nodal irradiation or addition and duration of ADT. While the basic groupings have remained intact over the past quarter-century, these categories have fissured further over the past two decades. Very low risk and very high risk groups have been added (13, 14), and the intermediate risk category has been subdivided into favorable and unfavorable groups (15). While the latter of these distinctions is also derived from a retrospective analysis of patients treated with radiotherapy, the NCCN guidelines have adopted this distinction to determine whether ADT should be incorporated into treatment. In spite of the field’s current reliance on this system, it has only been validated as prognostic, rather than predictive, in determining patient outcomes.

Importantly, while risk stratification of patients with other cancers has begun to incorporate genetic testing and other molecular biomarker assays, biological information has not yet penetrated the AJCC Staging or NCCN risk stratification systems for patients with prostate cancer. There are now multiple commercially available assays that utilize molecular biomarkers (e.g. Decipher^®^, Prolaris^®^, Oncotype DX^®^ Score) as well as another that uses artificial intelligence (ArteraAI Prostate). While none of these tests are able predict response to therapy, they have all been shown to be prognostic (16, 17), and there is considerable interest in incorporating them into risk stratification and treatment selection algorithms. For instance, NRG Oncology is currently coordinating two randomized trials (NRG GU-009 and GU-010) which are evaluating of the Decipher^®^ assay to either intensify or de-intensify concomitant ADT depending on the prognostic score (18, 19). All of these tests, however, are expensive, and may require several weeks to obtain results after the initial histopathologic diagnosis of prostate cancer has been issued.

In the present analysis, we demonstrate the possible utility of simple, biopsy-based biomarker analysis in patients who are undergoing SBRT for treatment of prostate cancer. To our knowledge, this is the first report assessing the impact of PTEN and ERG abnormalities in patients undergoing prostate SBRT. Despite the relatively small number of patients available for analysis (n = 97), clear detriments in both bPFS and OS were observed in this patient cohort, particularly for patients with abnormalities in both PTEN and ERG. Furthermore, tumor mutational status appeared to be comparable, if not slightly more powerful, in estimating risk of biochemical failure than NCCN risk groupings. With the exception of substantial comorbidities as determined by the CCI, dual abnormality in PTEN and ERG was the factor most associated with poor overall survival.

There are several limitations to the current analysis. First, it is limited by its retrospective nature and a relatively small number of patients, though PTEN and ERG abnormalities were strongly associated with oncologic outcomes despite the sample size. Despite the inclusion of only patients treated with SBRT, there was some heterogeneity in treatment with a minority of patients receiving pelvic radiotherapy as a component of their therapy. However, these variations were consistent across the groups, without significant changes in distribution when stratified by mutational status. Of those patients treated to the prostate and proximal seminal vesicles only, roughly half received a nominal prescription dose of 3500 cGy while the remainder received 3625 cGy. While unlikely, it is possible that an unquantified benefit is conferred by the higher dose. Nonetheless, it is of note that all patients were treated with robotic SBRT on the CyberKnife platform, which is inherently inhomogeneous as compared to linear accelerator based SBRT and typically yields mean prostate doses that substantially exceed the nominal prescription dose. Furthermore, a recent large multi-institutional analysis of nearly two thousand patients demonstrated no difference in oncologic outcomes between these two regimens (20).

In summary, these relatively simple, biopsy-based biomarkers are prognostic for prostate cancer patients undergoing SBRT treatment. Each of these studies can be performed with immunohistochemistry (IHC) (21), which is both less expensive and requires fewer resources than other genetic tests such as fluorescence in-situ hybridization or microarray technology (22). Their ease of use and relatively low cost may make them attractive options to help guide treatment decisions in this patient population. Further research in the prospective setting is warranted to further study these biomarkers and their potential role in guiding therapeutic decision making in this patient population.

## Conclusions

ERG rearrangements and PTEN deletions detected on biopsy samples are associated with poorer oncologic outcomes, including both bPFS and OS, in prostate cancer patients treated with SBRT. Given their relative ease and cost, these biomarkers merit further study in a large trial their utility for risk stratification and treatment selection.

## Data availability statement

The raw data supporting the conclusions of this article will be made available by the authors to qualified investigators, without undue reservation.

## Ethics statement

The studies involving humans were approved by Georgetown University Institutional Review Board. The studies were conducted in accordance with the local legislation and institutional requirements. The participants provided their written informed consent to participate in this study.

## Author contributions

MR: Formal Analysis, Investigation, Methodology, Visualization, Writing – original draft, Writing – review & editing. TS: Formal Analysis, Methodology, Writing – review & editing. AZ: Data curation, Formal Analysis, Methodology, Writing – review & editing. MD: Data curation, Methodology, Writing – review & editing. MA: Data curation, Writing – review & editing. TY: Data curation, Writing – review & editing. SL: Writing – review & editing. BC: Writing – review & editing. DK: Writing – review & editing. SS: Data curation, Methodology, Writing – review & editing. RH: Writing – review & editing. AK: Writing – review & editing. SC: Conceptualization, Data curation, Investigation, Methodology, Supervision, Writing – review & editing.
